# Clinical and laboratory studies of the novel cyclin-dependent kinase inhibitor dinaciclib (SCH 727965) in acute leukemias

**DOI:** 10.1007/s00280-013-2249-z

**Published:** 2013-08-15

**Authors:** Ivana Gojo, Mariola Sadowska, Alison Walker, Eric J. Feldman, Swaminathan Padmanabhan Iyer, Maria R. Baer, Edward A. Sausville, Rena G. Lapidus, Da Zhang, Yali Zhu, Ying-Ming Jou, Jennifer Poon, Karen Small, Rajat Bannerji

**Affiliations:** 1 Division of Hematology/Oncology, Marlene and Stewart Greenebaum Cancer Center, University of Maryland, Baltimore, MD USA; 2 Translational Core Laboratory, Marlene and Stewart Greenebaum Cancer Center, University of Maryland, Baltimore, MD USA; 3 Division of Hematology, The Ohio State University, Columbus, OH USA; 4 Division of Hematology and Medical Oncology, Cornell Medical Center, New York, NY USA; 5 The Institute for Drug Development, CTRC, University of Texas Health Science Center, San Antonio, TX USA; 6 Merck Sharp & Dohme Corp., Whitehouse Station, NJ USA; 7 Present Address: The Sidney Kimmel Comprehensive Cancer Center at Johns Hopkins, The Bunting Blaustein Clinical Research Building, 1650 Orleans Street, Baltimore, MD 21287 USA; 8 Present Address: The Methodist Cancer Center, Weill Cornell Medical School, Houston, TX USA; 9 Present Address: Novartis Pharmaceuticals Corporation, East Hanover, NJ USA; 10 Present Address: The Cancer Institute of New Jersey, Robert Wood Johnson Medical School, New Brunswick, NJ USA

**Keywords:** Dinaciclib, Acute leukemia, Cyclin-dependent kinase, CDK inhibitor, Mcl-1

## Abstract

**Purpose:**

Dinaciclib inhibits cyclin-dependent kinases 1, 2, 5, and 9 with a better therapeutic index than flavopiridol in preclinical studies. This study assessed the activity of dinaciclib in acute leukemia both in the clinic and in vitro.

**Methods:**

Adults with relapsed/refractory acute myeloid leukemia (*n* = 14) and acute lymphoid leukemia (*n* = 6) were treated with dinaciclib 50 mg/m^2^ given as a 2-h infusion every 21 days.

**Results:**

Most patients had dramatic but transient reduction in circulating blasts; however, no remissions were achieved on this schedule. The most common toxicities were gastrointestinal, fatigue, transaminitis, and clinical and laboratory manifestations of tumor lysis syndrome, including one patient who died of acute renal failure. Dinaciclib pharmacokinetics showed rapid (2 h) achievement of maximum concentration and a short elimination/distribution phase. Pharmacodynamic studies demonstrated in vivo inhibition of Mcl-1 expression and induction of PARP cleavage in patients’ peripheral blood mononuclear cells 4 h after dinaciclib infusion, but the effects were lost by 24 h and did not correlate with clinical outcome. Correlative in vitro studies showed that prolonged exposures to dinaciclib, at clinically attainable concentrations, result in improved leukemia cell kill.

**Conclusions:**

While dinaciclib given as a 2-h bolus did not exhibit durable clinical activity, pharmacokinetic and pharmacodynamic data support the exploration of prolonged infusion schedules in future trials in patients with acute leukemias.

**Electronic supplementary material:**

The online version of this article (doi:10.1007/s00280-013-2249-z) contains supplementary material, which is available to authorized users.

## Introduction

Uncontrolled proliferation and deregulated cell cycle progression are hallmarks of many human cancers, including acute leukemias. Cyclin-dependent kinases (CDKs) are serine/threonine kinases that primarily regulate the mammalian cell cycle and thus represent an appealing therapeutic target [1–3]. However, progression through the cell cycle is a complex process that depends on synchronized activity of different CDKs (CDK1, CDK2, CDK4, CDK6), their phosphorylation status, and association with cyclins and endogenous Cip/Kip or INK4 inhibitors [4–7]. Alteration in any of these components may lead to cell cycle arrest and/or apoptosis or, as seen in cancer cells, to unregulated cellular proliferation [2, 3]. Besides controlling cell cycle, several members of CDK family have specific cell cycle-independent effects. CDK5 is known to play a role in neuronal function and cellular senescence [8], while CDK7/cyclin H and CDK9/cyclin T complexes modulate cellular transcription through phosphorylation of the COOH-terminal domain (CTD) of RNA polymerase II [9–12]. Inhibition of transcriptional CDKs may induce cell death through down-regulation of several short-lived proteins, including the anti-apoptotic proteins Mcl-1 and XIAP [13–16].

Given the key role of CDKs in both cell cycle regulation and cellular transcription processes which are frequently altered in malignant cells, several small-molecule CDK inhibitors with a broad but not completely overlapping CDK inhibitory activity have been developed and tested clinically as anti-cancer agents (flavopiridol, SNS-032, seliciclib, AT7519, PHA-793887) [17, 18]. Flavopiridol was the first in this class to enter the clinical arena and has shown promising single-agent clinical activity in patients with high-risk chronic lymphocytic leukemia (CLL), but also toxicity associated with its narrow therapeutic index [19, 20]. While flavopiridol displayed limited single-agent activity in patients with acute leukemia [21], when given in timed sequence with cytarabine and mitoxantrone (FLAM regimen), an approach based on pre-clinical in vitro modeling in leukemia cells [22], direct anti-leukemia cytotoxicity and encouraging clinical responses were observed in patients with poor-risk newly diagnosed and relapsed/refractory acute myeloid leukemia (AML) [23–26].

Dinaciclib is a novel potent CDK1, CDK2, CDK5, and CDK9 inhibitor (IC_50_ 1–4 nM) that was found to have a superior therapeutic index (TI; maximum tolerated dose to effective dose) in comparison both SNS-032 and flavopiridol (TI of 10 vs. 2 vs. <1, respectively) in a pre-clinical in vivo screen using an A2780 ovarian carcinoma xenograft mouse model [27]. Compared with flavopiridol, dinaciclib appears to be an equally potent inhibitor of CDK1 and CDK9, but a much stronger inhibitor of CDK2 and CDK5, and more selective in an in vitro kinase screen [27]. Preclinical studies have also demonstrated that dinaciclib is a potent growth inhibitor (median IC_50_, 11 nM) and inducer of apoptosis in a variety of human cell lines, including leukemia cells and xenograft models [27–30]. Based on these findings and the direct cytotoxic effect of flavopiridol in vivo, we hypothesized that dinaciclib might be useful to treat patients with acute leukemias.

Two phase I clinical studies explored the activity of dinaciclib given as a 2-h infusion on days 1, 8, 15 every 28 days [31] or once every 21 days [32] to patients with solid tumors. The recommended phase II doses (RPTD) were 12 and 50 mg/m^2^, respectively. The primary dose-limiting toxicities (DLT) were sepsis, hyperuricemia, and hypotension with the weekly schedule and neutropenia and transient liver function test (LFT) abnormalities with the every 21-day schedule. Based on these toxicity profiles, the latter dose/schedule was selected for testing in acute leukemia patients. We report our initial clinical experience with dinaciclib in patients with advanced AML and acute lymphoid leukemia (ALL), along with correlative pharmacokinetic (PK) and pharmacodynamic studies. We also present observations prompted by our clinical and pharmacological findings that, in turn, will inform further development of dinaciclib in acute leukemias.

## Materials and methods

### Study design and patients

This was a phase II multi-center study (NCT00798213) of dinaciclib that was originally designed as a randomized study for relapsed and/or refractory AML patients comparing the efficacy of dinaciclib versus gemtuzumab ozogamicin (GO, Mylotarg) and a single-arm study of dinaciclib in patients with ALL. AML patients treated with GO could cross over to dinaciclib at the time of disease progression. The study was approved by the Institutional Review Board (IRB) at each participating institution and was conducted in accordance with the Declaration of Helsinki and in compliance with International Conference on Harmonization Good Clinical Practice Guidelines. Written informed consent was obtained from all patients before study enrollment.

Patients with CD33-expressing AML (≥60 years old) or with ALL (≥18 years old) in first or second relapse or having primary refractory disease or refractory disease after first salvage and not considered candidates for hematopoietic stem cell transplantation (HSCT) were eligible for the study, provided they had an Eastern Cooperative Oncology Group (ECOG) performance status of ≤1, white blood cell (WBC) count ≤30 × 10^9^/L, serum creatinine ≤2 mg/dL, alanine aminotransferase (ALT) or aspartate aminotransferase (AST) ≤2.5 X upper limit of normal and normal bilirubin. Patients were ineligible if they had active central nervous system disease, a second malignancy requiring treatment, known HIV, hepatitis B or hepatitis C infection, previous GO or CDK inhibitor treatment, or previous HSCT.

This study was initiated on March 10, 2009. Results were analyzed as of June 21, 2011. The study was terminated after enrollment of 26 patients due to change in the study sponsor; however, a total of 20 patients received dinaciclib (2 patients crossed over from GO), and the clinical experience with dinaciclib in these patients is presented below.

### Study objectives and treatment plan

The primary objective was to determine the overall response rate (ORR), and secondary objectives included time to progression (TTP), safety, dinaciclib PKs, and exploratory biomarker studies.

Dinaciclib 50 mg/m^2^ was given as a 2-h intravenous (IV) infusion in cycle 1, and if well tolerated, intra-patient dose escalation to 70 mg/m^2^ was allowed in cycle 2. Each cycle lasted 21 days. Patient could continue treatment on the study in the absence of disease progression or unacceptable toxicity. The next cycle of therapy could start if non-hematologic toxicity from previous cycle recovered to ≤grade 1, and dose delay of up to 3 weeks was allowed for recovery. Permanent dose reduction was required for any ≥grade 3 clinically significant non-hematologic adverse event (70 mg/m^2^ → 50 mg/m^2^; 50 mg/m^2^ → 40 mg/m^2^; 40 mg/m^2^ → 30 mg/m^2^; 30 mg/m^2^ → discontinue). To allow for continued treatment of subjects whose cytopenias were due to leukemia and not due to drug toxicity, subsequent cycles of treatment were allowed if absolute neutrophil count (ANC) ≥0.5 × 10^9^/L or for ANC <0.5 × 10^9^/L but no more than 20 % below the baseline pretreatment levels and platelet count ≥20 × 10^9^/L or platelets <20 × 10^9^/L but no more than 20 % below the baseline pretreatment levels. Anti-emetic prophylaxis including serotonin-receptor antagonist, metoclopramide, or prochloperazine with or without dexamethasone was allowed. After 10 patients had been treated with dinaciclib, the protocol was amended to require hospitalization for cycle 1 treatment, with administration of pre-treatment rasburicase, allopurinol, hydration, phosphate binders, and early administration of Kayexalate (sodium polystyrene sulfonate) for hyperkalemia, including pre-treatment for potassium ≥4 mmol/L. Intense monitoring of laboratory parameters for evidence of tumor lysis syndrome was required, including repeat measurements of serum potassium at 1, 2, 3, 6, and 24 h after start of dinaciclib infusion or more frequently if needed. Dexamethasone 20 mg IV was given early if symptoms of cytokine release syndrome were observed [19].

### Response and toxicity evaluation

Response was assessed by bone marrow (BM) examination on day 21 of cycles 1 and 2, and every 6 weeks thereafter until complete remission or progressive disease (PD). Response was determined according to the revised recommendations of the International Working Group (IWG) for AML [33]. Toxicities were graded according to the National Cancer Institute Common Terminology Criteria for Adverse Events (NCI CTCAE), version 3.0 (http://ctep.cancer.gov/protocolDevelopment/electronic_applications/docs/ctcaev3.pdf). Hematologic and chemistry profiles were monitored on day 1 and 2 of each cycle or until resolution of acute toxicities and subsequently at least once a week or more frequently depending on the individual patient’s needs.

### Pharmacokinetic analysis

Blood samples for PK evaluations (3 mL each) were collected into prechilled K2 EDTA tubes at 0, 0.25–0.5, 1–2, and 4 h after the end of the 2-h infusion on day 1 of cycles 1, 2, and 3. Samples were kept on ice and centrifuged within 1 h of collection at 2,700*g*/4 °C for 10 min; separated plasma was immediately frozen on dry ice and then stored at −20 °C or below.

Dinaciclib plasma concentrations were determined using a validated high-performance liquid chromatography assay with tandem mass spectrometry detection [34]. The internal standard was [13C3]-dinaciclib. The lower limit of quantitation (LLOQ) was 0.0250 ng/mL and the upper limit of quantitation was 10.0 ng/mL. The accuracy at the LLOQ was −1.0 and the precision coefficient of variation (CV) was 10.6 %. The accuracy at low, medium, and high quality control (QC) concentration was 5.8, 5.7, and 2.7, respectively, and precision at low, medium, and high QC concentrations was 4.9, 4.0, and 4.2, respectively. Dilution integrity was demonstrated at 10,000 ng/mL.

Plasma concentration versus time profiles and systemic exposure of dinaciclib in this study were estimated using a population pharmacokinetic (PPK) model-based approach. A 2-compartment PPK model was developed with data from two phase I studies in solid tumors [31, 32]. The model fits were conducted using NONMEM V, level 1.1 (GloboMax LLC), and a first-order conditional estimation method was employed for all model runs. The model included between-subject variability on clearance and volume of distribution, and mixed error terms.

### Pharmacodynamic analysis on primary patient’ samples

Peripheral blood (PB) samples, ≈20 cc/time point, were collected pre-treatment, and 4 and 24 h after the end of the dinaciclib infusion in cycle 1 in patients who had PB blasts ≥3 × 10^9^/L for whom a laboratory facility was available for immediate specimen processing. For Western blot analysis, PB mononuclear cells (PBMC) were isolated by gradient centrifugation using lymphocyte separation medium (LSM) (Mediatech Inc., Manassas, VA, USA), and protein extracts were prepared and analyzed for the expression of Mcl-1, phospho-Rb (Ser807/811), and PARP cleavage as described below.

### In vitro studies on primary leukemia cells and human leukemia cell lines

Primary leukemia cells for in vitro studies were isolated from BM or PB obtained from AML or ALL patients enrolled on a separate University of Maryland, Baltimore, IRB-approved protocol for tissue collection and not treated on this clinical study. The cells were isolated by gradient centrifugation using LSM (Mediatech) according to the manufacturer’s protocol. Cells were used either fresh or after viable freezing (FBS/10 % DMSO). Primary leukemia cells were cultured in RPMI 1640 (Invitrogen, Grand Island, NY, USA) supplemented with 15 % heat-inactivated FBS (HyClone Laboratories Inc., Red Bank, NJ, USA) and 200 mM l-Glutamine (Invitrogen). Leukemia cell lines HL-60 (AML), MOLT-4 (T cell ALL), and K562 (chronic myelogenous leukemia [CML] in erythroid blast transformation) were purchased from American Type Culture Collection (ATCC, Manassas, VA, USA) and cultured in RPMI 1640 (Invitrogen) supplemented with 10 % heat-inactivated FBS (HyClone Laboratories Inc.) and 200 mM l-Glutamine (Invitrogen).

Dinaciclib (SCH727965) was obtained from Merck & Co. Inc., Whitehouse Station, NJ, USA (originally Schering-Plough, Kenilworth, NJ, USA). The drug was dissolved in DMSO at 20 mM, aliquoted, and stored at −20 °C until use. Z-VAD-FMK (BD Parmingen, San Diego, CA, USA), a pancaspase inhibitor, was dissolved in DMSO at 20–60 mM and stored in aliquots at −20 °C until use.

Cell proliferation after dinaciclib treatment was assessed using the WST-1 cell proliferation assay (Roche, Indianapolis, IN, USA). Primary leukemia cells were cultured in the presence of increasing concentrations of the dinaciclib (0.0004–10 μM) for 24 h. Leukemia cells lines (HL-60, MOLT-4, K562) were cultured in the presence of increasing concentrations of the dinaciclib (0.0004–10 μM) for 2–72 h. Cells cultured in the presence of an equivalent volume of DMSO served as a treatment control. 4–8 replicates were plated for each drug dilution or control. The IC_50_ values for the WST-1 assay were calculated using GraphPad Prism 5 software (La Jolla, CA, USA).

Apoptosis assay was performed on fresh primary leukemia cells and leukemia cell lines (HL-60 and K562) treated with dinaciclib (2–200 nM) or an equivalent volume of DMSO (control) for different lengths of time (2–24 h). After treatment, cells were washed twice with cold PBS, resuspended in 1X binding buffer, and stained with propidium iodide (PI) and Annexin V using a FITC Annexin V Apoptosis Detection Kit I (BD Pharmingen) according to the manufacturer’s instructions. The stained cells were evaluated within 1 h on a FACScan (BD Biosciences, San Jose, CA, USA) and analyzed using FlowJo software (Tree Star, Ashland, OR, USA).

For cell cycle analysis, leukemia cells (HL-60 and K562) were treated with dinaciclib (20 and 200 nM) for 2–24 h, pelleted, fixed in 70 % ethanol at −20 °C, stained with 500 μl PI/RNase staining buffer (BD Pharmingen), and analyzed using a FACScan (BD Bioscience) and FlowJo software (Tree Star).

For Western blot analysis, leukemia cell pellets (primary leukemia cells, HL-60, MOLT-4, K562) were washed with PBS and lysed with RIPA buffer (Sigma-Aldrich, St. Louis, MO, USA) supplemented with Complete Mini™ protease inhibitor and PHOStop™ phosphatase inhibitors (Roche). Equal amounts of proteins up to 25 μg were separated on 4–12 % NuPAGE precast gels in 1X MOPS or 1X MES buffer (Invitrogen) and electro-blotted to PVDF membranes (Millipore, Billerica, MA, USA). The membranes were blocked with 5 % dry milk in 1X TBS/0.1 % Tween 20 for at least 1 h at room temperature. Blots were incubated with primary antibody (Ab) overnight at 4 °C, washed 3 times in TBS/0.1 % Tween 20, and then incubated with secondary Ab for 1 h at room temperature. Blots were washed three times, and the signal was detected with Amersham™ ECL™ detection systems (GE Healthcare, Pittsburgh, PA, USA). The following primary Abs were used: mouse anti-PARP (BD Pharmingen), rabbit anti-phospho-Rb (Ser807/811) and rabbit anti-Mcl-1 (both from Santa Cruz, Santa Cruz, CA, USA), rabbit anti-caspase 9 p35 and anti-caspase 3 (both from Cell Signaling, Danvers, MA, USA), and mouse anti-β-actin (Sigma-Aldrich). Commercially available HL-60 cell extract, non-induced SW-101 and etoposide-induced SW-102, were used as negative and positive controls for PARP cleavage (Enzo Life Sciences, Plymouth Meeting, PA, USA). Secondary horseradish peroxidase-conjugated anti-mouse or anti-rabbit Abs were from Cell Signaling (Beverly, MA, USA). The Western blot signals were measured by densitometry using ImageJ 1.45I software (NIH, Bethesda, USA).

### Statistics

The primary efficacy endpoint of the study was the ORR, including complete remission (CR), CR with incomplete count recovery (CRi), and partial remission. The secondary efficacy endpoint was TTP, defined as the time from the date of randomization (AML)/enrollment (ALL)/first dose of dinaciclib (AML crossed over from GO) to the first date of documented PD or death due to PD.

## Results

### Patient characteristics

Between March 2009 and April 2010, a total of 26 patients were enrolled on this phase II study. Twenty patients with advanced acute leukemia received dinaciclib (2 patients crossed over from GO) and their characteristics are detailed in Table 1. Most patients treated with dinaciclib had AML (70 %), were 60 years or older (85 %), and had disease refractory to prior therapies (80 %). Data on 8 patients treated with GO and statistical analyses on their outcomes are presented in the Online Tables S1 and S2a–c.

**Table 1:** Characteristics of patients treated with dinaciclib

Total no. of patients	20
Age, years	
Median	70
Range	38–76
Age ≥60 years	
No. (%)	17 (85)
Diagnosis	
AML, no. (%)	14 (70)
ALL, no. (%)	6 (30)
Gender	
Male, no. (%)	14 (70)
Female, no. (%)	6 (30)
ECOG performance status	
0	5
1	14
2	1
Race	
White	19
Black	1
*Karyotype*	
AML	
Complex (≥3 chromosomal abnormalities)	4
Monosomy 7	2
Trisomy 8	2
Other chromosomal abnormalities	3
Normal karyotype	3
ALL	
*t*(9;22)	2
Other chromosomal abnormalities	3
Normal karyotype	1
Prior chemotherapy regimens	
Median	1
Range	1–4
Disease status	
First relapse	4
Primary refractory	11
Refractory disease after salvage	5
Median WBC count ×10^6^/L (range)	2,650 (400–17,200)
Median circulating blast count ×10^6^/L (range)	627 (0–9,975)
Median bone marrow blast count, % (range)	54 (1.4–96)

### Toxicities

A total of 38 cycles of dinaciclib were given to 20 patients, with a median of 1 cycle per patient (range, 1–5). Eight of 10 patients were given dinaciclib 70 mg/m^2^ in cycle 2, while one patient received dinaciclib 40 mg/m^2^ and another dinaciclib 50 mg/m^2^ in cycle 2 because of syncope and cytokine release syndrome, respectively, in cycle 1. Dinaciclib was dose-reduced to 50 mg/m^2^ in cycle 3 in an additional patient because of fatigue. Overall, 15 (75 %) patients treated with dinaciclib experienced grade ≥3 treatment-related adverse events (AE) with hematologic toxicities and fatigue being the most frequent. Though difficult to assess in a leukemia patient population, the most common dinaciclib-related ≥grade 3 hematologic toxicities included leukopenia (40 %), anemia (25 %), febrile neutropenia (25 %), thrombocytopenia (20 %), and neutropenia (10 %). Non-hematologic treatment-related adverse events (AE) occurring in ≥10 % patients are presented in Table 2. The most common non-hematologic toxicities were gastrointestinal, fatigue, and laboratory abnormalities, including electrolyte disturbances and transient transaminitis. Grade 1 and 2 diarrhea was common, occurring in 70 % of patients, with onset within hours after initiation of treatment but usually resolved within a day. Grade ≥3 fatigue was noted in 4 (20 %) patients, while syncope that resolved without intervention was observed in the same patient in cycles 1 and 2. Overall, half of the patients had at least one significant tumor lysis syndrome (TLS)-associated laboratory abnormality (defined by Cairo-Bishop criteria) [35] in cycle 1, including hyperphosphatemia (30 %), hypocalcemia (25 %), hyperkalemia (25 %), and/or hyperuricemia (10 %). Three patients had clinical grade ≥3 TLS, manifesting as cardiac arrhythmia (1) and/or renal insufficiency (2), including a patient with AML who developed hyperacute TLS requiring hemodialysis and died of acute renal failure (ARF) on day 13 of cycle 1, despite intense pre-treatment prophylaxis with rasburicase, calcium acetate, and sevelamer and aggressive treatment of TLS with insulin, calcium gluconate, and sodium polystyrene sulfonate. This patient had evidence of TLS even before treatment such as abnormal electrolytes, elevated creatinine, including history of chronic renal insufficiency, and required hydroxyurea for count control before study treatment. We examined the association between the baseline and pre-treatment (day 1) measurements of total WBC count, peripheral blood blast count, % blast in the BM, uric acid, lactate dehydrogenase (LDH), and creatinine on the development of TLS. Despite the small sample size, we found that higher pre-treatment creatinine might be associated with the increased risk of developing TLS (Logistic Regression Wald test; p value = 0.0543). An additional 9 patients died on study, 8 from PD and 1 from intracranial hemorrhage due to disease-related thrombocytopenia. Of the remaining 10 patients, 9 were removed from study because of PD and 1 per patient request.

**Table 2:** Dinaciclib-related non-hematologic adverse events occurring in ≥2 (10 %) patients

Adverse events	Cycle 1 *n* = 20		Cycle 2 *n* = 10		Cycle 3 *n* = 6	
	All (%)	Grade ≥3	All (%)	Grade ≥3	All (%)	Grade ≥3
Cardiovascular						
Hypertension	1 (5)	−	−	−	1 (17)	−
Hypotension	6 (30)	1	2 (20)	−	−	−
Sinus Tachycardia	2 (10)	−	−	−	−	−
Constitutional						
Cytokine release sy	2 (10)	−	−	−	1 (17)	1
Dehydration	1 (5)	1	1 (10)	−	−	−
Fatigue	6 (30)	2	2 (20)	1	1 (17)	1
Gastrointestinal						
Anorexia	1 (5)	−	−	−	1 (17)	−
Diarrhea	14 (70)	1	7 (70)	1	2 (33)	−
Nausea	8 (40)	1	3 (30)	−	1 (17)	−
Stomatitis	1 (5)	−	1 (10)	1	−	−
Vomiting	9 (45)	−	2 (20)	−	2 (33)	−
Hepatic						
Hyperbilirubinemia	2 (10)	−	1 (10)	−	−	−
Increased ALT	4 (20)	−	1 (10)	−	1 (17)	−
Increased AST	8 (40)	3	1 (10)	−	−	−
Infections						
Bacteremia	2 (10)	2	−	−	−	−
Pneumonia	2 (10)	2	1 (10)	−	−	−
Metabolic and electrolyte abnormalities						
Hyperglycemia	4 (20)	2	−	−	−	−
Hyperkalemia	3 (15)	2	1 (10)	−	−	−
Hyperphosphatemia	5 (25)	1	1 (10)	−	−	−
Hypoalbuminemia	4 (20)	−	1 (10)	−	−	−
Hypocalcemia	6 (30)	1	−	−	−	−
Hypokalemia	2 (10)	−	−	−	−	−
Hyponatremia	2 (10)	1	−	−	−	−
Increased LDH	2 (10)	2	−	−	−	−
Tumor lysis sy	3 (15)	3	−	−	−	−
Neurology						
Syncope	1 (5)	1	1 (10)	1	−	−
Renal						
Acute renal failure	2 (10)	2	−	−	−	−
Increased creatinine	3 (15)	1	−	−	−	−

### Clinical outcomes

**Fig. 1:**
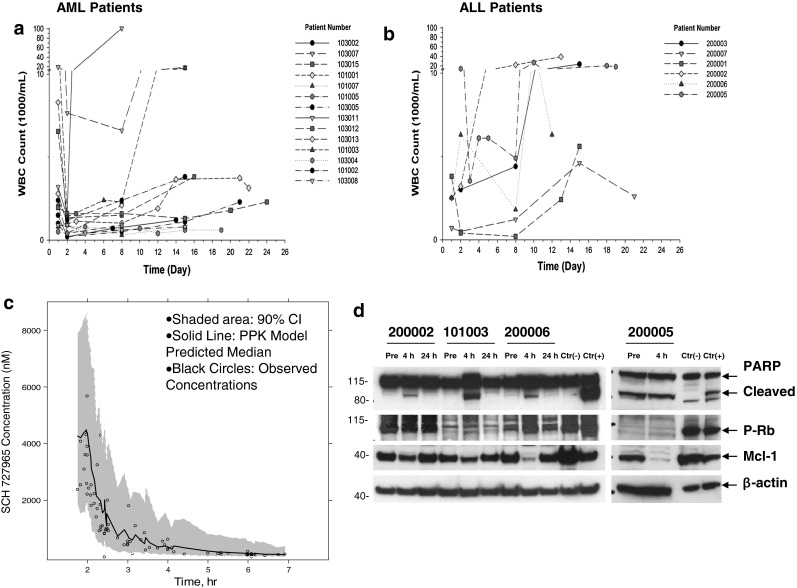
The effects of dinaciclib on circulating WBCs, pharmacokinetics, and pharmacodynamics. **a** Graph demonstrating rapid decline in the WBC count in AML patients after treatment with dinaciclib, followed by gradual recovery, usually by day 15. **b** Graph demonstrating rapid decline in the WBC count in ALL patients after treatment with dinaciclib. **c** Population pharmacokinetic (PPK) model-based prediction of plasma concentration versus time profiles following a single 2-h infusion of 50 mg/m^2^ dinaciclib. **d** Western blot analysis of protein lysates obtained from PBMC collected prior to and at 4 and 24 h after dinaciclib infusion to measure the expression of p-Rb and Mcl-1 and induction of PARP cleavage. β-actin controls are shown to document equivalent loading and transfer of proteins. *Lanes* were loaded with 8–25 μg of protein

No objective responses by IWG criteria were documented, but evidence of direct anti-leukemia cytotoxic activity manifesting as a temporary decrease in PB and/or marrow blasts was observed in 60 % of patients. Ten of 13 (77 %) patients with circulating blasts (7/7 AML; 3/6 ALL) had >50 % decrease in the absolute blast count (ABC) within 1–8 days of the first dinaciclib dose, including 6 (46 %) with >80 % decrease. The median pretreatment WBC count for all patients was 2,650 × 10^6^/L (range, 400–17,200 × 10^6^/L) and it declined to 1,045 × 10^6^/L (range, 200–14,000 × 10^6^/L) and 1,350 × 10^6^/L (range, 200–100,800 × 10^6^/L) on days 2 and 8, respectively. The median duration of the WBC nadir was 5 (range, 0–7) days. The decline in WBC count during cycle 1 in patients with AML and ALL is presented in Fig. 1a, b, respectively. For patients with circulating blasts, the median pre-treatment absolute blast count (ABC) was 1,130 × 10^6^/L (range, 220–9,975 × 10^6^/L), the median ABC nadir was 200 × 10^6^/L (range, 0–1,350 × 10^6^/L), the median time to nadir was 3 (range, 1–16) days, and the duration of nadir was 7 (range, 1–24) days. Overall, 8 of 13 patients who had follow-up BM evaluations had an average of 36 % (range, 6–67 %) reduction in BM blasts in the course of treatment, with 2 patients achieving >50 % blast reduction in the marrow (1 on day 21 and 1 on day 42).

For AML patients included in the initial randomization, median TTP was 5.14 (range, 1.57–13.14) weeks, and for ALL patients, TTP ranged from 1 to 8.14 weeks. Two AML patients who crossed over from GO had TTP of 7.86 and 9.14 weeks, respectively.

### Pharmacokinetics

PK data were available from all 20 patients. Observed plasma concentrations are summarized in Table 3. Plasma concentrations predicted from the PPK model correlated with the observed values. Additionally, the model parameter estimates with and without inclusion of data from the current study are similar. This indicates that the PPK model-based approach can be used to estimate the pharmacokinetics of dinaciclib in this study.

**Table 3:** Mean (CV %) plasma concentrations of dinaciclib 2-h infusion (cycle 1–3)

Dose	Plasma concentration (ng/mL)^a^				
	40 mg/m^2^ (cycle 2, *n* = 1)	50 mg/m^2^ (cycle 1, *n* = 20; cycle 2, *n* = 1; cycle 3, *n* = 2)		70 mg/m^2^ (cycle 2, *n* = 8; cycle 3, *n* = 3)	
Time^b^ (hours)	Mean (ng/mL)	Mean (ng/mL)	CV %	Mean (ng/mL)	CV %
2	1,010	1,130	39	1,700	48
2.25	1,100	707	68	724	20
2.5	NA	354	52	457	87
3	NA	195	55	307	22
4	118	139	47	125	32
6	42.4	36.7	41	52.2	40

*CV* coefficient of variation, *NA* not applicable (no sample collected)

^a^Combined data from cycles 1, 2, and 3 for each dose level

^b^Time after the initiation of the dinaciclib infusion

As shown in Fig. 1c, the observed plasma concentrations collected at sparse sampling times are within 90 % confidence interval of model-predicted values. Thus, based on the model-predicted PK profile, the estimated mean AUC values at doses of 50 mg/m^2^ and 70 mg/m^2^ are 3,556 ng h/mL (CV% = 15) and 4,979 ng h/mL, respectively. The AUC values estimated in this study are similar to those observed previously in phase I studies [31, 32].

### Pharmacodynamics

Pre-treatment, 4- and 24-h post-end of infusion samples of PBMC were obtained from 1 AML and 3 ALL patients. We examined the in vivo effects of dinaciclib on the inhibition of serine 807 and 811 phosphorylation of the Rb protein, as a readout for CDK1 and CDK2 inhibition, and on the expression of Mcl-1, a short-lived anti-apoptotic protein, as an indirect measure of the ability of dinaciclib to repress transcription through the inhibition of CDK9. We also examined whether treatment with dinaciclib leads to induction of PARP cleavage, indicating apoptotic cell death. As depicted in Fig. 1d, decrease in Mcl-1 was seen in all specimens at 4-h post-treatment, concordant with inhibition of CDK9, but the expression of Mcl-1 returned to baseline by 24 h, suggesting that the inhibition was lost by that time. Induction of PARP cleavage was also observed at 4 h, although in one specimen PARP cleavage product was high at baseline. Decline in p-Rb was observed in 1 patient, while 2 patients had almost undetectable p-Rb levels at baseline.

### In vitro studies on primary leukemia cells

The clinical and pharmacodynamic data prompted further studies in leukemia cell lines and primary leukemia cells. As presented in Online Fig. S1a–c, we were able to demonstrate that prolonged exposures (24 or 72 vs. 2 h) to dinaciclib concentrations attainable in vivo have a more potent growth inhibitory and apoptosis-inducing effect across different leukemia cell lines (HL-60, MOLT-4, K562). We have also demonstrated that dinaciclib down-regulates the expression of the anti-apoptotic protein Mcl-1 independently of caspases and induces PARP cleavage in leukemia cell lines; however, these effects were most prominent with prolonged exposures (6 and 24 h) (Online Fig. S2a–b). A 2-h exposure to dinaciclib was sufficient to induce down-regulation of Mcl-1 and PARP cleavage only in the HL-60 cell line; interestingly, and in agreement with in vivo data, this effect was lost by 24 h. Down-regulation of p-Rb was also observed in 2 of 3 cell lines.

**Fig. 2:**
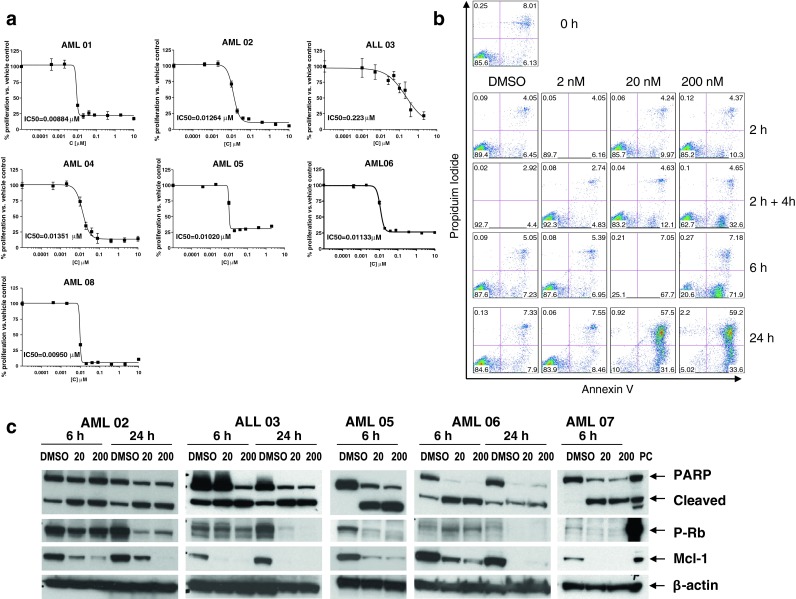
Prolonged exposure to dinaciclib effectively inhibits proliferation, induces apoptosis, and down-regulates Mcl-1 and phospho-Rb expression in primary leukemia cells. **a** Six primary AML samples were exposed to DMSO (control) or dinaciclib (0.0004–10 μM) for 24 h and the IC_50_ values were estimated using the WST-1 assay. **b** Primary AML cells (AML 08) were exposed to dinaciclib (2, 20, and 200 nM) for the indicated times and the extent of cell death was monitored by measuring Annexin V/PI staining by flow cytometry. **c** Five primary AML and ALL samples were exposed to DMSO (control) and dinaciclib (20 and 200 nM) for 6 and/or 24 h, after which whole cells were lysed and proteins extracted, and subjected to Western blot analysis to measure the expression of PARP, Mcl-1, p-Rb. β-actin controls are shown to document equivalent loading and transfer of proteins. *Lanes* were loaded with 8–25 μg of protein

Thus, we next examined the effect of prolonged dinaciclib exposure on primary leukemia cells. Figure 2a demonstrates that treatment with dinaciclib for 24 h produced similar growth inhibitory effects across primary AML samples with different cytogenetic and molecular findings (Table 4) as measured by the WST-1 assay, with IC_50_ values in the range of those observed for leukemia cell lines (IC_50_ 0.008–0.017 μM) and achievable in vivo. Dinaciclib induced apoptosis in primary AML cells (AML 08) in a time- and concentration-dependent manner, as measured by flow cytometric analysis of AnnexinV/PI staining (Fig. 2b). Similarly, down-regulation of Mcl-1 and induction of PARP cleavage were observed in all 5 primary leukemia samples after 6-h exposure to dinaciclib (≥20 nM), but more prominent effects were noted at the 200 nM concentration and with 24-h exposure (Fig. 2c). The effects of dinaciclib on p-Rb expression were less consistent. While in 3 of 5 primary cell samples dinaciclib treatment resulted in down-regulation of p-Rb at 6 h, in 2 of the samples the level of p-Rb remained the same (AML 02) or even increased (AML 06), followed by complete inhibition of p-Rb at 24 h. Thus, data with cell lines and primary leukemia cells are consistent and strongly suggest that prolonged exposures to dinaciclib, at clinically attainable concentrations, should result in improved leukemia cell kill.

**Table 4:** Characteristics of primary leukemia cells for in vitro studies

Patient samples	Diagnosis	Disease status	Cytogenetics	FLT3	NPM1	Source	% Blast
AML 01	AML arising from MDS	Status post-azacitidine for MDS	*t*(3;21), +8	WT	ND	BM	8
AML 02	AML post-ET	Untreated	Del (20q), *t*(1;19) (q25;q13.3)	WT	WT	BM	21
ALL 03	ALL	First relapse	Normal	ND	ND	PB	60
AML 04	AML	Primary refractory	Normal	WT	WT	BM	25
AML 05	AML	Untreated	Inv(16)	WT	WT	BM	61
AML 06	AML	Untreated	Normal	D835	MUT	PB	90
AML 07	AML	Untreated	Normal	ITD	ND	BM	78
AML 08	AML	Untreated	Normal	WT	MUT	BM	73

*FLT3* FMS-like tyrosine kinase 3, *NMP1* nucleophosmin 1, *MDS* myelodysplastic syndrome, *ET* essential thrombocytosis, *WT* wild type, *ND* not done, *ITD* internal tandem duplication, *MUT* mutated, *BM* bone marrow, *PB* peripheral blood

## Discussion

Single-agent dinaciclib 50 mg/m^2^ given as a 2-h infusion has potent, but short-lived, cytoreductive activity, without objective responses observed in our patients with advanced acute leukemia. Dinaciclib administration was associated with frequent occurrence of diarrhea and tumor lysis manifestations, suggesting that dinaciclib shares the toxicity profile of other CDK inhibitors in leukemia patients [21, 23–26, 36]. Pharmacodynamic data demonstrated a rapid decrease in Mcl-1 protein levels and induction of PARP cleavage in patients’ peripheral blood leukemia cells upon dinaciclib treatment, but with quick recovery once dinaciclib level declines. Further, in vitro studies on leukemia cells suggest that longer exposures to dinaciclib, at clinically achievable concentrations, have a potent cytotoxic effect. Prolonged infusion schedules of dinaciclib administration should be explored in future trials in acute leukemia.

In this study, we observed >50 % reduction in the circulating blast count within 8 days of dinaciclib administration in 77 % of patients; however, the nadir was short (≤1 week). The effect of dinaciclib on the BM blast content was modest, but, due to the timing of follow-up marrow examination, we might have missed the maximum effect. Despite difficulties in comparing phase I/II studies with different agents, single-agent dinaciclib appears to have cytoreductive activity similar to that of flavopiridol. Flavopiridol administered on a hybrid schedule [21] or as 1-h bolus [25] for 3 days produced a >50 % decrease in WBC count in approximately 83 and 44 % of acute leukemia patients, respectively, but the short duration of the nadir and the lack of measurable BM response to single-agent flavopiridol were similar to our experience with dinaciclib [21].

The side effects observed after dinaciclib administration in this study appear very similar to those described for CLL patients receiving dinaciclib on a weekly schedule [37] and resemble toxicities seen with flavopiridol in leukemia patient populations [21, 23–26]. There was no difference between the 50 and 70 mg/m^2^ doses. The most frequent toxicities included transient gastrointestinal toxicities, LFT abnormalities, fatigue, and hypotension. Electrolyte abnormalities associated with tumor lysis were noted in at least 50 % of patients, with 3 patients developing clinical TLS, including one who died of acute renal failure. Thus, intense monitoring of laboratory parameters and early intervention to abate the manifestations of TLS are necessary for the safe administration of this drug to acute leukemia patients.

We performed limited PK analysis in this study; however, the partial PK profile observed in our patients appears similar to those obtained in studies with more extensive PK profiling [31, 32, 34] and can be predicted using the PPK approach. Overall, dinaciclib exhibits linear PK, with dose-related (50 → 70 mg/m^2^) increases in exposure (Cmax, AUC) and moderate inter-patient variability. The Cmax was achieved within 2 h and, following the end of infusion, dinaciclib was rapidly eliminated. Our pharmacodynamic Western blot studies showed that in vivo administration of dinaciclib led to down-regulation of Mcl-1 (34–76 %) and induction of PARP cleavage in PBMC sampled 4 h following the end of the infusion in all 4 specimens examined, but Mcl-1 levels returned to baseline at 24 h, indicating that the surviving cells were able to recover. Down-regulation of Mcl-1 was also observed after dinaciclib treatment in CLL patients [37]; however, while serving as a pharmacodynamic marker of CDK inhibition, Mcl-1 modulation was not found to correlate with response in CLL patients [38]. Although data suggest that complete suppression of p-Rb upon dinaciclib treatment correlates closely with induction of apoptosis in solid tumor cell lines and xenograft models [27–29], we could not consistently demonstrate such a relationship between p-Rb inhibition and cell death in patients’ PBMC in vivo or in primary leukemia cells or cell lines treated in vitro, including at the times when Mcl-1 down-regulation was clearly present. This discrepancy may, in part, reflect heterogeneity of p-Rb expression in different leukemia cells, but also suggests that, at least in some leukemias, the primary target of dinaciclib activity may not be CDK1/2. A similar observation was recently made using dinaciclib in rhabdomyosarcoma tumor xenograft models [30]. Thus, whether inhibition of CDK1/2 or CDK9 or both is required for dinaciclib activity is likely to depend on cellular context.

Given that 2-h dinaciclib infusion had only transient effect on peripheral blast count, and given the observed pharmacokinetic and pharmacodynamic data, we performed in vitro studies on leukemia cells and demonstrated that prolonged exposures to dinaciclib (≥24 h), at concentrations (≤200 nM) that are far below the Cmax (>4,000 nM) observed in the clinical study, caused more profound and persistent down-regulation of Mcl-1 and/or p-Rb across all cell lines/primary leukemia cells, leading to more effective cell kill. Preliminary PK data from clinical studies in solid tumors, using 8- and 24-h continuous infusion, suggest that average dinaciclib concentrations of 200–1,031 nM (7.4–50 mg/m^2^) and 82.3–184 nM (7.4–14.6 mg/m^2^) can be achieved, respectively [32]. Based on our in vitro data, these drug concentrations should be effective in killing leukemia cells.

With the exception of CLL [19, 20], single-agent CDK inhibitors have demonstrated very modest clinical anti-cancer activity in a broad range of tumors [17]. As shown in this study, dinaciclib has direct anti-leukemia cytotoxicity but effects are of short duration, suggesting that it needs to be combined with chemotherapy or other novel agents whose activity may be enhanced by dinaciclib-induced down-regulation of Mcl-1 in acute leukemias. Combination strategies also need to take into account the cell cycle-modulating impact of dinaciclib; this approach has been effective for flavopiridol given in timed sequence with cytarabine and mitoxantrone in leukemia patients [22–26]. The observation that longer exposure times produce more effective cell kill in vitro and the effectiveness of weekly dinaciclib administration in CLL [37] support pharmacologically driven studies that may optimize dinaciclib both as a single agent and as part of combination strategies in acute leukemias.

## Electronic supplementary material

Below is the link to the electronic supplementary material.

**Fig. S1** Dinaciclib induces inhibition of leukemia cell proliferation and time-dependent apoptosis in human leukemia cell lines (WST-1 assay, flow cytometry-Annexin V/PI and PI staining) (PDF 176 kb)

**Fig. S2** Treatment of leukemia cell lines with dinaciclib leads to Mcl-1 and phospho-Rb down-regulation and induction of PARP cleavage that is cell type-, time- and concentration- dependent (Western blot analysis) (PDF 215 kb)

**Table S1.** Characteristics of AML patients treated with gemtuzumab ozogamicin (DOC 34 kb)

**Table S2a-c.** Response rates and median time to progression in all treated AML patients (gemtuzumab ozogamicin vs. dinaciclib) (DOCX 18 kb)

## References

[1] Malumbres M, Barbacid M (2009). Cell cycle, CDKs and cancer: a changing paradigm. Nat Rev Cancer.

[2] Sausville EA (2002). Complexities in the development of cyclin-dependent kinase inhibitor drugs. Trends Mol Med.

[3] Shapiro GI (2006). Cyclin-dependent kinase pathways as targets for cancer treatment. J Clin Oncol.

[4] Pines J (1994). The cell cycle kinases. Semin Cancer Biol.

[5] Sherr CJ (1994). G1 phase progression: cycling on cue. Cell.

[6] Morgan DO (1995). Principles of CDK regulation. Nature.

[7] Sherr CJ, Roberts JM (1999). CDK inhibitors: positive and negative regulators of G1-phase progression. Genes Dev.

[8] Dhariwala FA, Rajadhyaksha MS (2008). An unusual member of the Cdk family: Cdk5. Cell Mol Neurobiol.

[9] Garriga J, Grana X (2004). Cellular control of gene expression by T-type cyclin/CDK9 complexes. Gene.

[10] Marshall NF, Peng J, Xie Z, Price DH (1996). Control of RNA polymerase II elongation potential by a novel carboxyl-terminal domain kinase. J Biol Chem.

[11] Meinhart A, Kamenski T, Hoeppner S, Baumli S, Cramer P (2005). A structural perspective of CTD function. Genes Dev.

[12] Oelgeschlager T (2002). Regulation of RNA polymerase II activity by CTD phosphorylation and cell cycle control. J Cell Physiol.

[13] Chen R, Keating MJ, Gandhi V, Plunkett W (2005). Transcription inhibition by flavopiridol: mechanism of chronic lymphocytic leukemia cell death. Blood.

[14] Gojo I, Zhang B, Fenton RG (2002). The cyclin-dependent kinase inhibitor flavopiridol induces apoptosis in multiple myeloma cells through transcriptional repression and down-regulation of Mcl-1. Clin Cancer Res.

[15] Kitada S, Zapata JM, Andreeff M, Reed JC (2000). Protein kinase inhibitors flavopiridol and 7-hydroxy-staurosporine down-regulate antiapoptosis proteins in B-cell chronic lymphocytic leukemia. Blood.

[16] Lam LT, Pickeral OK, Peng AC, Rosenwald A, Hurt EM, Giltnane JM, Averett LM, Zhao H, Davis RE, Sathyamoorthy M, Wahl LM, Harris ED, Mikovits JA, Monks AP, Hollingshead MG, Sausville EA, Staudt LM (2001) Genomic-scale measurement of mRNA turnover and the mechanisms of action of the anti-cancer drug flavopiridol. Genome Biol 2(10):RESEARCH0041 10.1186/gb-2001-2-10-research0041 PMC57796 11597333

[17] Cicenas J, Valius M (2011). The CDK inhibitors in cancer research and therapy. J Cancer Res Clin Oncol.

[18] Massard C, Soria JC, Anthoney DA, Proctor A, Scaburri A, Pacciarini MA, Laffranchi B, Pellizzoni C, Kroemer G, Armand JP, Balheda R, Twelves CJ (2011). A first in man, phase I dose-escalation study of PHA-793887, an inhibitor of multiple cyclin-dependent kinases (CDK2, 1 and 4) reveals unexpected hepatotoxicity in patients with solid tumors. Cell Cycle.

[19] Lin TS, Ruppert AS, Johnson AJ, Fischer B, Heerema NA, Andritsos LA, Blum KA, Flynn JM, Jones JA, Hu W, Moran ME, Mitchell SM, Smith LL, Wagner AJ, Raymond CA, Schaaf LJ, Phelps MA, Villalona-Calero MA, Grever MR, Byrd JC (2009). Phase II study of flavopiridol in relapsed chronic lymphocytic leukemia demonstrating high response rates in genetically high-risk disease. J Clin Oncol.

[20] Phelps MA, Lin TS, Johnson AJ, Hurh E, Rozewski DM, Farley KL, Wu D, Blum KA, Fischer B, Mitchell SM, Moran ME, Brooker-McEldowney M, Heerema NA, Jarjoura D, Schaaf LJ, Byrd JC, Grever MR, Dalton JT (2009). Clinical response and pharmacokinetics from a phase 1 study of an active dosing schedule of flavopiridol in relapsed chronic lymphocytic leukemia. Blood.

[21] Blum W, Phelps MA, Klisovic RB, Rozewski DM, Ni W, Albanese KA, Rovin B, Kefauver C, Devine SM, Lucas DM, Johnson A, Schaaf LJ, Byrd JC, Marcucci G, Grever MR (2010). Phase I clinical and pharmacokinetic study of a novel schedule of flavopiridol in relapsed or refractory acute leukemias. Haematologica.

[22] Karp JE, Ross DD, Yang W, Tidwell ML, Wei Y, Greer J, Mann DL, Nakanishi T, Wright JJ, Colevas AD (2003). Timed sequential therapy of acute leukemia with flavopiridol: in vitro model for a phase I clinical trial. Clin Cancer Res.

[23] Karp JE, Blackford A, Smith BD, Alino K, Seung AH, Bolanos-Meade J, Greer JM, Carraway HE, Gore SD, Jones RJ, Levis MJ, McDevitt MA, Doyle LA, Wright JJ (2010). Clinical activity of sequential flavopiridol, cytosine arabinoside, and mitoxantrone for adults with newly diagnosed, poor-risk acute myelogenous leukemia. Leuk Res.

[24] Karp JE, Passaniti A, Gojo I, Kaufmann S, Bible K, Garimella TS, Greer J, Briel J, Smith BD, Gore SD, Tidwell ML, Ross DD, Wright JJ, Colevas AD, Bauer KS (2005). Phase I and pharmacokinetic study of flavopiridol followed by 1-beta-d-arabinofuranosylcytosine and mitoxantrone in relapsed and refractory adult acute leukemias. Clin Cancer Res.

[25] Karp JE, Smith BD, Levis MJ, Gore SD, Greer J, Hattenburg C, Briel J, Jones RJ, Wright JJ, Colevas AD (2007). Sequential flavopiridol, cytosine arabinoside, and mitoxantrone: a phase II trial in adults with poor-risk acute myelogenous leukemia. Clin Cancer Res.

[26] Karp JE, Smith BD, Resar LS, Greer JM, Blackford A, Zhao M, Moton-Nelson D, Alino K, Levis MJ, Gore SD, Joseph B, Carraway H, McDevitt MA, Bagain L, Mackey K, Briel J, Doyle LA, Wright JJ, Rudek MA (2011). Phase 1 and pharmacokinetic study of bolus-infusion flavopiridol followed by cytosine arabinoside and mitoxantrone for acute leukemias. Blood.

[27] Parry D, Guzi T, Shanahan F, Davis N, Prabhavalkar D, Wiswell D, Seghezzi W, Paruch K, Dwyer MP, Doll R, Nomeir A, Windsor W, Fischmann T, Wang Y, Oft M, Chen T, Kirschmeier P, Lees EM (2010). Dinaciclib (SCH 727965), a novel and potent cyclin-dependent kinase inhibitor. Mol Cancer Ther.

[28] Fu W, Ma L, Chu B, Wang X, Bui MM, Gemmer J, Altiok S, Pledger WJ (2011). The cyclin-dependent kinase inhibitor SCH 727965 (dinacliclib) induces the apoptosis of osteosarcoma cells. Mol Cancer Ther.

[29] Feldmann G, Mishra A, Bisht S, Karikari C, Garrido-Laguna I, Rasheed Z, Ottenhof NA, Dadon T, Alvarez H, Fendrich V, Rajeshkumar NV, Matsui W, Brossart P, Hidalgo M, Bannerji R, Maitra A, Nelkin BD (2011). Cyclin-dependent kinase inhibitor Dinaciclib (SCH727965) inhibits pancreatic cancer growth and progression in murine xenograft models. Cancer Biol Ther.

[30] Gorlick R, Kolb EA, Houghton PJ, Morton CL, Neale G, Keir ST, Carol H, Lock R, Phelps D, Kang MH, Reynolds CP, Maris JM, Billups C, Smith MA (2012). Initial testing (stage 1) of the cyclin dependent kinase inhibitor SCH 727965 (dinaciclib) by the pediatric preclinical testing program. Pediatr Blood Cancer.

[31] Nemunaitis J, Saltzman M, Rosenberg MA, Khaira D, Small K, Kirschmeier P, Statkevich P, Abutarif M, Yao S, Bannerji R (2009) A phase I dose-escalation study of the safety, pharmacokinetics, and pharmacodynamics of SCH 727965, a novel cyclin-dependent kinase inhibitor, administered weekly in subjects with advanced malignancies. J Clin Oncol 27:15s (suppl; abstr 3535)

[32] Mita MM, Mita AC, Moseley J, Poon J, Small KA, Jou Y, Kirschmeier P, Zhang D, Statkevich P, Sankhala KK, Sarantopoulos J, Cleary JM, Chirieac LR, Rodig S, Bannerji R, Shapiro GI (2011) A phase I study of the CDK inhibitor dinaciclib (SCH 727965) administered every 3 weeks in patients with advanced malignancies: final results. J Clin Oncol 29 (suppl; abstr 3080)

[33] Cheson BD, Bennett JM, Kopecky KJ, Buchner T, Willman CL, Estey EH, Schiffer CA, Doehner H, Tallman MS, Lister TA, Lo-Coco F, Willemze R, Biondi A, Hiddemann W, Larson RA, Lowenberg B, Sanz MA, Head DR, Ohno R, Bloomfield CD, International Working Group for Diagnosis SoRCTO, Reporting Standards for Therapeutic Trials in Acute Myeloid L (2003) Revised recommendations of the International Working Group for Diagnosis, Standardization of Response Criteria, Treatment Outcomes, and Reporting Standards for Therapeutic Trials in Acute Myeloid Leukemia. J Clin Oncol 21(24):4642–4649 10.1200/JCO.2003.04.036 14673054

[34] Zhang D, Mita M, Shapiro GI, Poon J, Small K, Tzontcheva A, Kantesaria B, Zhu Y, Bannerji R, Statkevich P (2012). Effect of aprepitant on the pharmacokinetics of the cyclin-dependent kinase inhibitor dinaciclib in patients with advanced malignancies. Cancer Chemother Pharmacol.

[35] Cairo MS, Bishop M (2004). Tumour lysis syndrome: new therapeutic strategies and classification. Br J Haematol.

[36] Blum KA, Ruppert AS, Woyach JA, Jones JA, Andritsos L, Flynn JM, Rovin B, Villalona-Calero M, Ji J, Phelps M, Johnson AJ, Grever MR, Byrd JC (2011). Risk factors for tumor lysis syndrome in patients with chronic lymphocytic leukemia treated with the cyclin-dependent kinase inhibitor, flavopiridol. Leukemia.

[37] Flynn JM, Jones JA, Andritsos L, Blum KA, Johnson AJ, Hessler J, Heerema NA, Wiley E, Poon J, Small KA, Jou Y, Zhang D, Statkevich P, Grever MR, Bannerji R, Byrd JC (2011) Phase I study of the CDK inhibitor dinaciclib (SCH 727965) in patients with relapsed/refractory CLL. J Clin Oncol 29 (suppl; abstr 6623)

[38] Woyach JA, Lozanski G, Ruppert AS, Lozanski A, Blum KA, Jones JA, Flynn JM, Johnson AJ, Grever MR, Heerema NA, Byrd JC (2012). Outcome of patients with relapsed or refractory chronic lymphocytic leukemia treated with flavopiridol: impact of genetic features. Leukemia.

