# Supplementary figures and images for: Clinical and laboratory studies of the novel cyclin-dependent kinase inhibitor dinaciclib (SCH 727965) in acute leukemias
Source: Cancer Chemother Pharmacol. 2013 Aug 15;72(4):897–908. doi: 10.1007/s00280-013-2249-z (PMC3784060; doi:10.1007/s00280-013-2249-z)

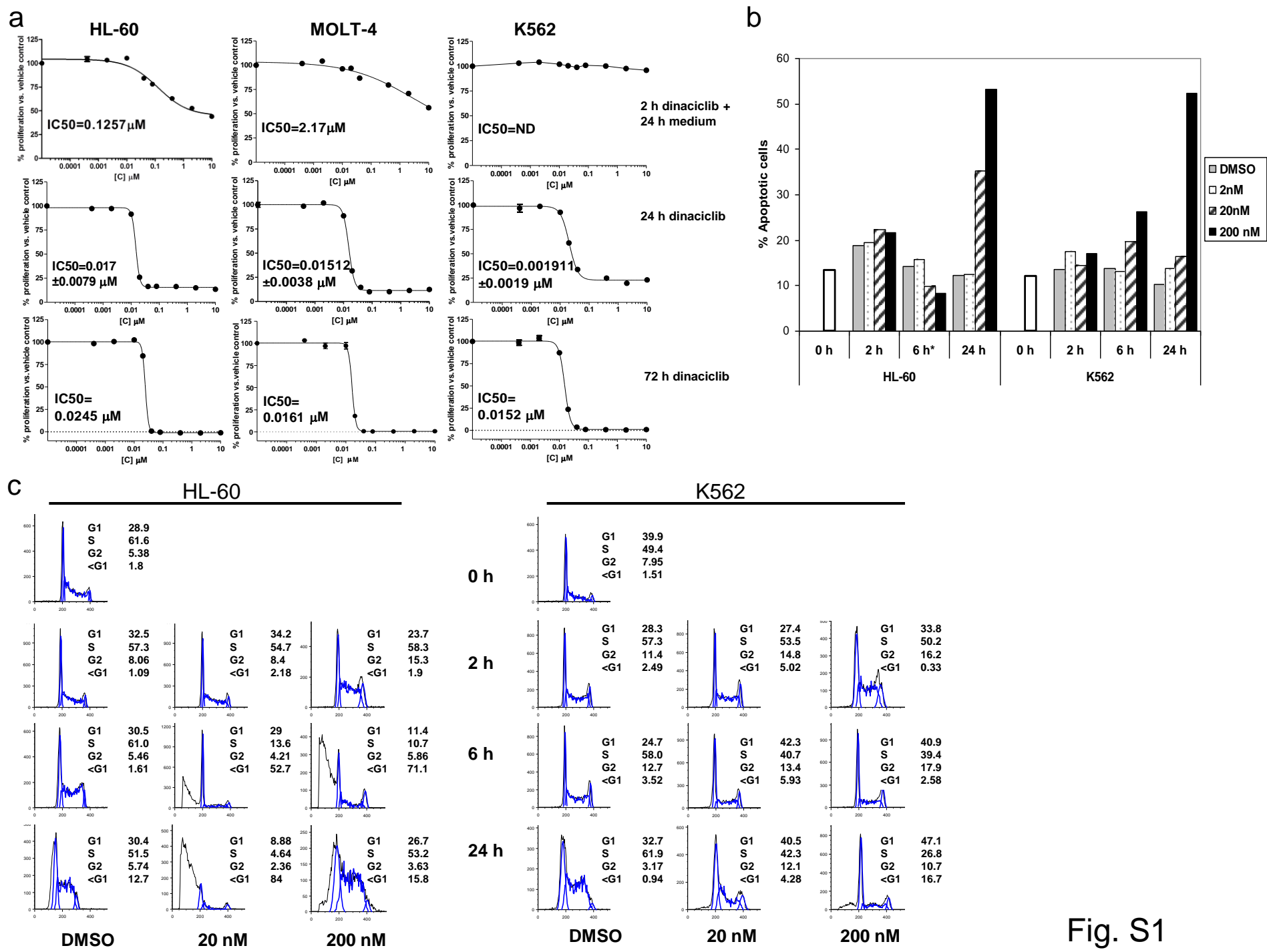

Supplement: Supplementary file 1 — Fig. S1 Dinaciclib induces inhibition of leukemia cell proliferation and time-dependent apoptosis in human leukemia cell lines (WST-1 assay, flow cytometry-Annexin V/PI and PI staining) (PDF 176 kb) [file 280_2013_2249_MOESM1_ESM.pdf]

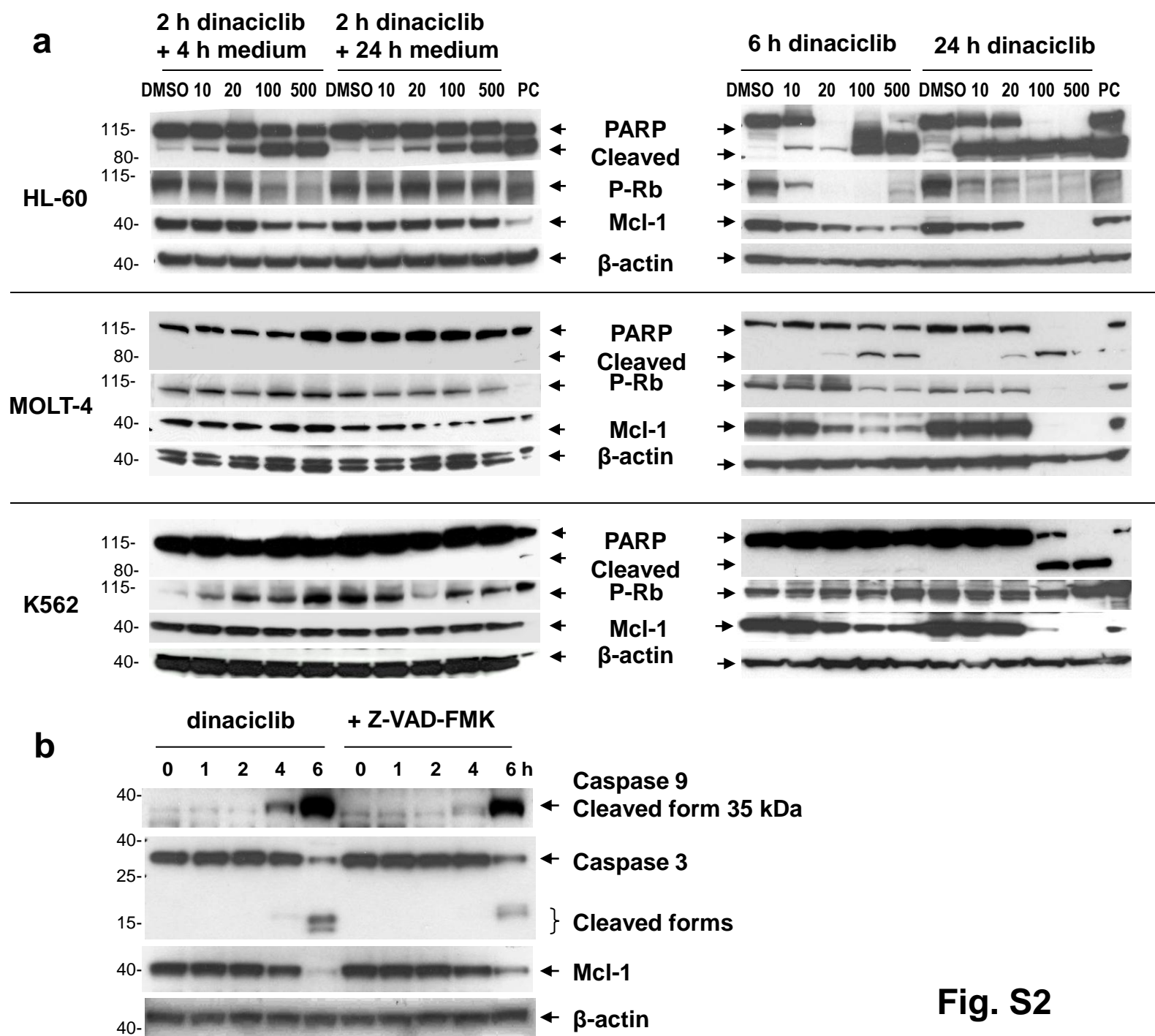

**Fig. S2**

Supplement: Supplementary file 2 — Fig. S2 Treatment of leukemia cell lines with dinaciclib leads to Mcl-1 and phospho-Rb down-regulation and induction of PARP cleavage that is cell type-, time- and concentration- dependent (Western blot analysis) (PDF 215 kb) [file 280_2013_2249_MOESM2_ESM.pdf]
