# Supplementary material for: Clinical and laboratory studies of the novel cyclin-dependent kinase inhibitor dinaciclib (SCH 727965) in acute leukemias
Source: Cancer Chemother Pharmacol. 2013 Aug 15;72(4):897–908. doi: 10.1007/s00280-013-2249-z (PMC3784060; doi:10.1007/s00280-013-2249-z)
Supplement: Supplementary file 3 — Table S1. Characteristics of AML patients treated with gemtuzumab ozogamicin (DOC 34 kb) [file 280_2013_2249_MOESM3_ESM.doc]

| **Table S1** Characteristics of AML patients treated with gemtuzumab ozogamicin | |
| --- | --- |
| Total No. of Patients | 8 |
| Age, years  Median  Range | 70  62-77 |
| Age ≥ 60 years  No. (%) | 8 (100) |
| Gender  Male, No. (%)  Female, No. (%) | 4 (50)  4 (50) |
| ECOG Performance Status  0  1  2 | 4  4  0 |
| Race  White | 8 |
| Karyotype  Complex (≥ 3 chromosomal abnormalities)  Monosomy 7  Trisomy 8  Normal karyotype  Unknown | 2  1  2  2  1 |
| Prior Chemotherapy Regimens  Median  Range | 1.5  1 - 2 |
| Disease Status  First relapse  Second Relapse  Primary refractory  Refractory disease after salvage | 2  1  4  1 |
| Median WBC count/µl (range) | 2600 (900 – 30400) |
| Median circulating blast count/µl (range) | 12.5 (0 – 20064) |
| Median bone marrow blast count, % (range) | 11.7 (5 – 90) |
