# Supplementary material for: Clinical and laboratory studies of the novel cyclin-dependent kinase inhibitor dinaciclib (SCH 727965) in acute leukemias
Source: Cancer Chemother Pharmacol. 2013 Aug 15;72(4):897–908. doi: 10.1007/s00280-013-2249-z (PMC3784060; doi:10.1007/s00280-013-2249-z)
Supplement: Supplementary file 4 — Table S2a-c. Response rates and median time to progression in all treated AML patients (gemtuzumab ozogamicin vs. dinaciclib) (DOCX 18 kb) [file 280_2013_2249_MOESM4_ESM.docx]

| **Table S2a**  Response rate in AML patients (all treated subjects), frequentist approach | | | | | | | |
| --- | --- | --- | --- | --- | --- | --- | --- |
|  |  | Number of | Number of | Observed | 2-sided 95% Confidence Interval | | |
| Treatment | N | Responders | Non-responders | Response Rate (%) | (Lower (%) | , | Upper (%)) |
| Dinaciclib | 12 | 0 | 12 | 0.000 | ( 0.000 | , | 26.465 ) |
| Mylotarg (GO) | 8 | 1 | 7 | 12.500 | ( 0.316 | , | 52.651 ) |
| Difference (SCH727965 - Mylotarg) | | | | -12.500 | ( -54.679 | , | 33.294 ) |

|  | | | | | | | | | | | | |
| --- | --- | --- | --- | --- | --- | --- | --- | --- | --- | --- | --- | --- |
| **Table S2b** Bayes estimate of response rate in AML patients (all treated subjects) | | | | | | | | | | | | |
|  |  | Beta Prior | |  | Number of |  | Beta Posterior | | | |  | Posterior Probability |
| Treatment |  | a | b | N | Responders |  | a | b | Mean* | StdDev |  | (This treatment is better) |
| Dinaciclib |  | 1 | 1 | 12 | 0 |  | 1 | 13 | 0.071 | 0.066 |  | 0.200 |
| Mylotarg (GO) |  | 1 | 3 | 8 | 1 |  | 2 | 10 | 0.167 | 0.103 |  | 0.801 |
| * This is Bayes estimate of the response rate. | | | | | | | | | | | | |
|  | | | | | | | | | | | | |

**Table S2c** Median time to progression and hazard ratio estimated based on all treated AML patients

| Treatment | Number of  Subjects | Number  Censored | Number of Events^a^ | Median (weeks) | | Hazard Ratio^b^ | | p-value |
| --- | --- | --- | --- | --- | --- | --- | --- | --- |
|  |  |  |  | Estimate | 95% CI | Estimate | 95% CI |  |
| Mylotarg (GO) | 8 | 1 | 7 | 7.57 | ( 2.71, 12.00) | 0.71 | ( 0.26, 1.95) | 0.5098 |
| Dinaciclib | 12 | 2 | 10 | 5.14 | ( 3.43, 9.14) |  |  |  |

^a^: Events = progression.

^b^: Hazard ratio is presented as Mylotarg/Dinaciclib.
